# Development of Foreign Mammary Epithelial Morphology in the Stroma of Immunodeficient Mice

**DOI:** 10.1371/journal.pone.0068637

**Published:** 2013-06-18

**Authors:** Gat Rauner, Amos Leviav, Eliezer Mavor, Itamar Barash

**Affiliations:** 1 Institute of Animal Science, ARO, The Volcani Center, Bet-Dagan, Israel; 2 The Robert H. Smith Faculty of Agriculture, Food and Environment, the Hebrew University of Jerusalem, Jerusalem, Israel; 3 Department of Plastic Surgery, Kaplan Medical Center, Rehovot, Israel; 4 Department of Surgery, Kaplan Medical Center, Rehovot, Israel; Garvan Institute of Medical Research, Australia

## Abstract

Systemic growth and branching stimuli, and appropriate interactions with the host stroma are essential for the development of foreign epithelia in the mammary gland of immunodeficient mice. These factors were manipulated to promote and investigate the generation of representative bovine epithelial morphology in the transplanted mouse mammary stroma. The bovine mammary epithelium is unique in its commitment to rapid proliferation and high rate of differentiation. Its morphological organization within a fibrotic stroma resembles that of the human breast, and differs significantly from the rudimentary ductal network that penetrates a fatty stroma in mice. Transplantation of bovine mammary epithelial cells into the cleared mammary fat pad of NOD-SCID mice led to continuous growth of epithelial structures. Multilayered hollow spheres developed within fibrotic areas, but in contrast to mice, no epithelial organization was formed between adipocytes. The multilayered spheres shared characteristics with the heifer gland’s epithelium, including lumen size, cell proliferation, cytokeratin orientation, estrogen/progesterone receptor expression and localization, and milk protein synthesis. However, they did not extend into the mouse fat pad via ductal morphology. Pre-transplantation of fibroblasts increased the number of spheres, but did not promote extension of bovine morphology. The bovine cells preserved their fate and rarely participated in chimeric mouse–bovine outgrowths. Nevertheless, a single case of terminal ductal lobuloalveolar unit (TDLU) development was recorded in mice treated with estrogen and progesterone, implying the feasibility of this representative bovine morphology’s development. *In vitro* extension of these studies revealed paracrine inhibition of bovine epithelial mammosphere development by adipocytes, which was also generalized to breast epithelial mammosphere formation. The rescue of mammosphere development by fibroblast growth factor administration evidences an active equilibrium between inhibitory and supportive effects exerted by the adipose and fibrotic regions of the stroma, respectively, which determines the development of foreign epithelium.

## Introduction

The mammalian mammary gland adopts a common tree-like morphology, with cyclic periods of production and regression. Hollow branches of epithelial origin stem from the nipple and penetrate the surrounding stroma. The branches are composed of an inner layer of luminal parenchymatic epithelial cells surrounded by an outer layer of myoepithelial cells that secrete the basal lamina separating the parenchyma from the stroma [1,2]. Lobular cells form secretory acinar structures at the end of each branch which, upon pregnancy and lactation, become alveolar cells that produce milk proteins. The mesenchymal stroma contains endothelial cells, extracellular matrix and inflammatory cells, but consists mainly of adipocytes and fibroblasts [3]. In addition to their supportive role, the latter maintain active interactions with the epithelia, which regulate developmental and functional activities such as branching and steroid paracrine signaling [4,5]. Importantly, the relative contents and interaction between the adipocytes and fibroblasts within the stroma differ among mammals. The mammary gland stroma of cattle is more fibrous and contains less adipose tissue than the fatty mouse mammary stroma [6]. Early partitioning of the adipose tissue by the connective tissue system is already observed in the neonate calf, in which the connective septa serve as paths for future extension of the epithelial structures. Consequently, fibrous stroma is present in both inter- and intralobular bovine mammary compartments [6,7]. The type of epithelial functional unit also differs among these species. In the virgin mouse, the terminus of the ductal network is generally composed of unbranched or minimally branched ductule with a single terminal endbud [8]. Conversely, the parenchyma of heifers develops as a ductal-lobular network and endbud structures are not present [9]. In these contexts, the morphology of the bovine gland resembles that of the human breast, in which the epithelium is generally closely associated with fibrous connective tissue [3,6,10], and branched terminal ductal lobuloalveolar units (TDLUs)—instead of the endbuds—represent the breast’s terminal epithelial unit [8]. These differences may be connected to the mechanisms regulating paracrine signaling, development and cell hierarchy in the mouse, bovine and human glands, which are the focus of this study. Ironically, our ability to study bovine or human cell hierarchy and development *in vivo* largely depends on xenotransplantation of foreign epithelium into the stroma of immunodeficient mice, and that requires functional interactions between these layers. Consequently, unlike the representative development and expansion of outgrowths from transplanted murine mammary epithelial cells (MECs) that fill the mouse’s cleared fat pad [11], transplantation of bovine or human MECs results in the morphological development of individual spherical structures with no extension [12,13]. For better harmony between the human epithelium and the endogenous stroma, Kuperwasser and colleagues [14] increased the fibrous component of the mouse stroma by pre- and co-transplantation of fibroblasts. This enabled the development of epithelial structures that were morphological representatives of the breast, upon organoid transplantation. Nevertheless, their expansion throughout the mouse fat pad has yet to be achieved, and the mechanism mediating the interactions with the mouse stroma has yet to be elucidated.

Introducing supportive conditions for the development of representative bovine morphological epithelial structures in the cleared mouse mammary fat pad is of high importance for the characterization of bovine mammary cell hierarchy and lineage commitment. It may also allow biotechnological attempts toward improving milk yield and livestock welfare via stem-cell manipulations [15,16]. In addition, the comparable morphology of the bovine gland and breast in terms of the functional epithelial unit and stromal composition makes the bovine mammary gland a more representative model of the human breast than the rodent mammary gland. Remarkably, despite this considerable similarity, there are almost no reports of mammary tumors in cows [17,18] and reviewed in [7], which is in striking contrast to the occurrence of breast cancer in humans. The reasons for the difference in mammary tumor prevalence between humans and bovines are unclear, and a better understanding of developmental processes in the bovine gland might shed light on this phenomenon.

Here we aimed to generate permissive conditions for the development of bovine mammary morphology in the mouse mammary stroma and to elucidate the reasons for its limited development in the foreign environment. Bovine (b) MECs were transplanted into the cleared mammary fat pad of immunodeficient NOD-SCID mice and were exposed to continuous administration of extraphysiological systemic steroid hormone levels or increased stromal fibrosis. The immediate microenvironment of the growing bovine epithelium was also a target for manipulation by co-transplantation of mouse (m) MECs [19]. We describe the contributions and limitations of each procedure with respect to intake rates, and the growth and development of distinct bovine morphological structures. Finally, the study was extended to an *in vitro* analysis of the factors mediating the inhibition of representative bovine epithelium development in a foreign environment and their relevance to human epithelial development. The data depict an active balance between the suppressive effect of the mouse fatty stroma and the protective effect of fibroblasts on the development of foreign epithelium. Basic characteristics of these effects are established with bovine and human epithelial mammospheres.

## Results

### Transplantation of dispersed bMECs into the mouse fat pad yields outgrowths that develop in confined fibrotic regions

**Figure 1:**
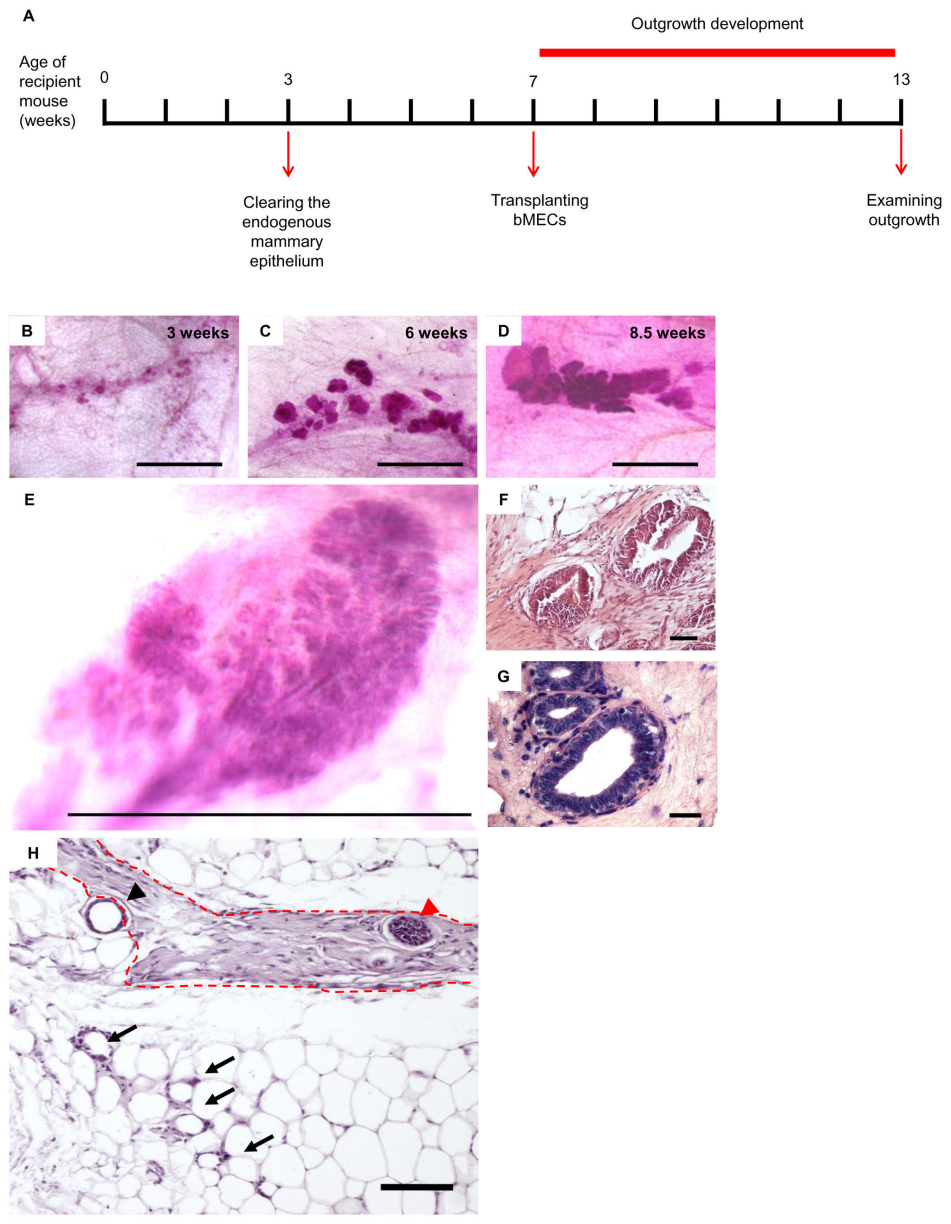
Transplanted bMECs generate developing spherical outgrowths in the mouse mammary fat pad with location-dependent morphology. **A**: Timeline of basic transplantation procedure. **B**–**D**: Carmine-stained wholemounts depicting typical outgrowth development at, respectively, 3, 6 and 8.5 weeks after cell transplantation. Bar = 1 mm. **E**: Carmine-stained wholemount of biopsy from the parenchymatic region of the heifer’s mammary gland. Bar = 1 mm. **F**: H&E-stained paraffin section from a Carmine-stained wholemount of 6-week-developed outgrowth. Bar = 50 µm. **G**: H&E-stained paraffin section from the parenchymatic region of a heifer’s mammary gland. Bar = 25 µm. **H**: H&E-stained paraffin section from a region containing outgrowths. A fibrous stromal area (dashed red line) encloses a solid, multilayered spherical outgrowth (red arrowhead) and partially encloses a hollow single-layered outgrowth (black arrowhead). Arrows indicate disorganized epithelial cells embedded in the adipose stroma. Bar = 100 µm.

A timeline was established for the prospective development of bMECs into epithelial structures within the host NOD-SCID mouse mammary stroma, and the monitoring of its consequences (Figure 1A). The timeline starts at the age of 3 weeks with the removal of the small rudimentary ductal network, thus clearing the fat pad of its parenchymatic constituent [20]. It proceeds with the transplantation of bMECs into the cleared mammary fat pad at the age of 7 weeks, and terminates 6 weeks later with an examination of the outgrowths at the age of 13 weeks.

Within 3 weeks of transplantation, outgrowths were already visible in the Carmine-stained fat pad wholemounts (Figure 1B). They occupied a distinct region, presumably the site of injection, and accumulated along dense, most likely fibrotic areas. The outgrowths were composed of separate, but adjacent, spherical epithelial structures that developed during the 6-week observation period, doubling in size approximately every week (Figure 1B–1D). Each structure might have been the progeny of a single or multiple repopulating cells. Regardless of their continuous growth, the spheres remained confined to a distinct region in the fat pad and did not penetrate the mouse adipose stroma in the way mouse-originated outgrowths do.

The morphology of the outgrowths that developed from the transplanted bMECs resembled the lobular morphology of the heifer’s mammary TDLU (Figure 1D and 1E). However, it lacked the duct-to-alveoli growth orientation characterizing the bovine mammary epithelium (Figure 1E). In fact, ductal structures were not observed.

Fat pad areas containing Carmine-stained outgrowths were removed under a binocular, and paraffin sections were prepared and stained with hematoxylin and eosin (H&E). Figure 1F reveals the structure of the outgrowths, which consisted of a multilayered epithelium surrounding a clear lumen, thus resembling the heifer’s mammary epithelial structure in both size and structure (Figure 1G). The multilayered outgrowths were almost exclusively found embedded in the fibrous stroma, where they maintained their developmental capability (Figure 1H). In contrast, small unorganized aggregates of cells were trapped between the adipocytes. These cells were likely transplanted bMECs, as this type of aggregation was not seen in the cleared fat pad. An intermediate morphological structure of single-layered outgrowths enclosing a lumen was frequently observed in the border between the adipose and fibrous stroma (Figure 1H).

### Outgrowths developed from transplanted bMECs proliferate, differentiate and express lineage markers

**Figure 2:**
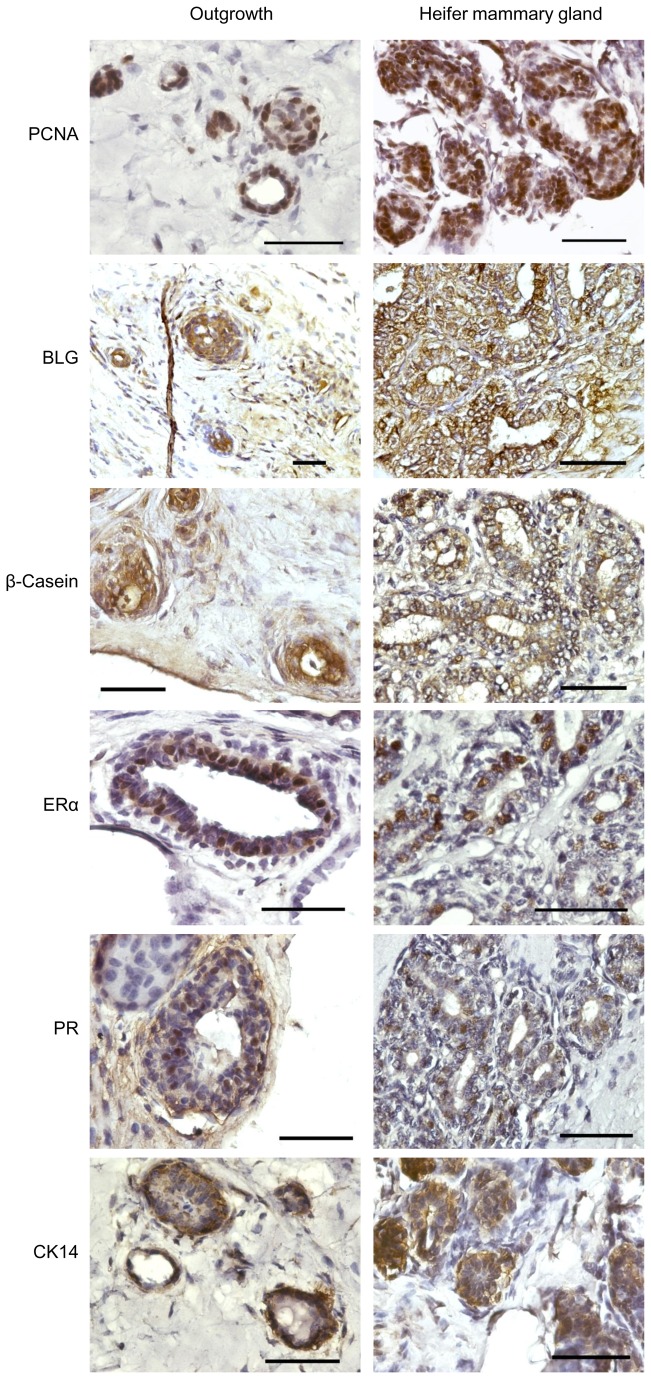
Bovine mammary outgrowths developed in the mouse, resemble the immunohistochemical characteristics of the donor tissue. Immunohistochemical analyses of selected markers on paraffin sections from bovine outgrowths (left panels) and mammary epithelium from heifer’s mammary gland (right panels). The outgrowths and the heifer’s tissue were subjected to similar histological procedures, including Carmine staining, prior to immunohistochemistry and hematoxylin counterstaining. Bar = 50 µm.

Outgrowths were further characterized by comparative immunohistochemical analyses of paraffin sections derived from the implanted mouse fat pad and the heifer’s mammary gland (Figure 2). Functional, lineage- and species-specific markers were examined. For reliable comparison, the heifer mammary wholemounts were stained with Carmine, including the concomitant treatments, similar to the implanted mouse fat pads. This caused significant tissue autofluorescence, which was avoided by using diaminobenzidine (DAB) for signal development.

PCNA immunostaining is an indicator of cell proliferation. Positive PCNA staining of bovine cells within the outgrowths resembled that observed in paraffin sections from the heifer’s gland. This indicated that cell proliferation accounts for the outgrowth’s increase in size over time, which subsequently yielded organized multilayered epithelial structures. Immunostaining of the bovine-specific β-lactoglobulin (BLG) milk protein as well as of β-casein revealed the presence of differentiated, or partially differentiated, luminal cells capable of producing these milk proteins, at least at a basal level, as seen in the heifer’s gland.

In mice, estrogen and progesterone mediate mammary ductal growth and branching, respectively, through their receptors in the mammary epithelial cells [21,22]. The role of estrogen and progesterone receptors (ERα and PR, respectively) in growth and morphogenesis of the bovine gland can be inferred from the effects resulting from manipulating the levels of their ligands [23]. Given the limited expansion of the transplanted outgrowths, we examined the expression patterns of ERα and PR on epithelial cells within the outgrowth compared with those in the heifer. Indeed, ERα expression in the outgrowths was confined to the mammary epithelial cells, recapitulating expression in the bovine donor where the ERα-expressing luminal cells do not reside in direct contact with the lumen but occupy a more basal layer [24]. Similarly, PR was also expressed in some of the cells within the inner luminal layer of both the heifer’s gland and the outgrowth structures.

More basal to the ERα- and PR-expressing cells and toward the stroma are the myoepithelial cells. These are characterized by CK14 expression in mouse and human glands [25,26]. Without exception, CK14 was located in this outer layer of the outgrowths, thus resembling its expression pattern in the bovine donor.

Collectively, these results indicate that the bovine outgrowths grow by cell proliferation and maintain an internal structure that resembles that in the heifer’s gland. This enables their functionality with respect to milk-protein expression.

Elevated levels of systemic estrogen and progesterone in the host mouse do not affect bovine outgrowth frequency. A unique observation of TDLU-like structure

Administration of extraphysiological levels of estrogen and progesterone is often used to mimic early pregnancy in mice due to their effects on ductal growth and branching, respectively. The role of ovaries and ovarian steroids in farm animals is less clear [7]. Nevertheless, short-term administration of extraphysiological doses of estrogen to heifers has been found to increase DNA synthesis by the mammary epithelium, [27], and of estrogen combined with progesterone to facilitate normal mammary growth in mature ovariectomized heifers [28].

**Figure 3:**
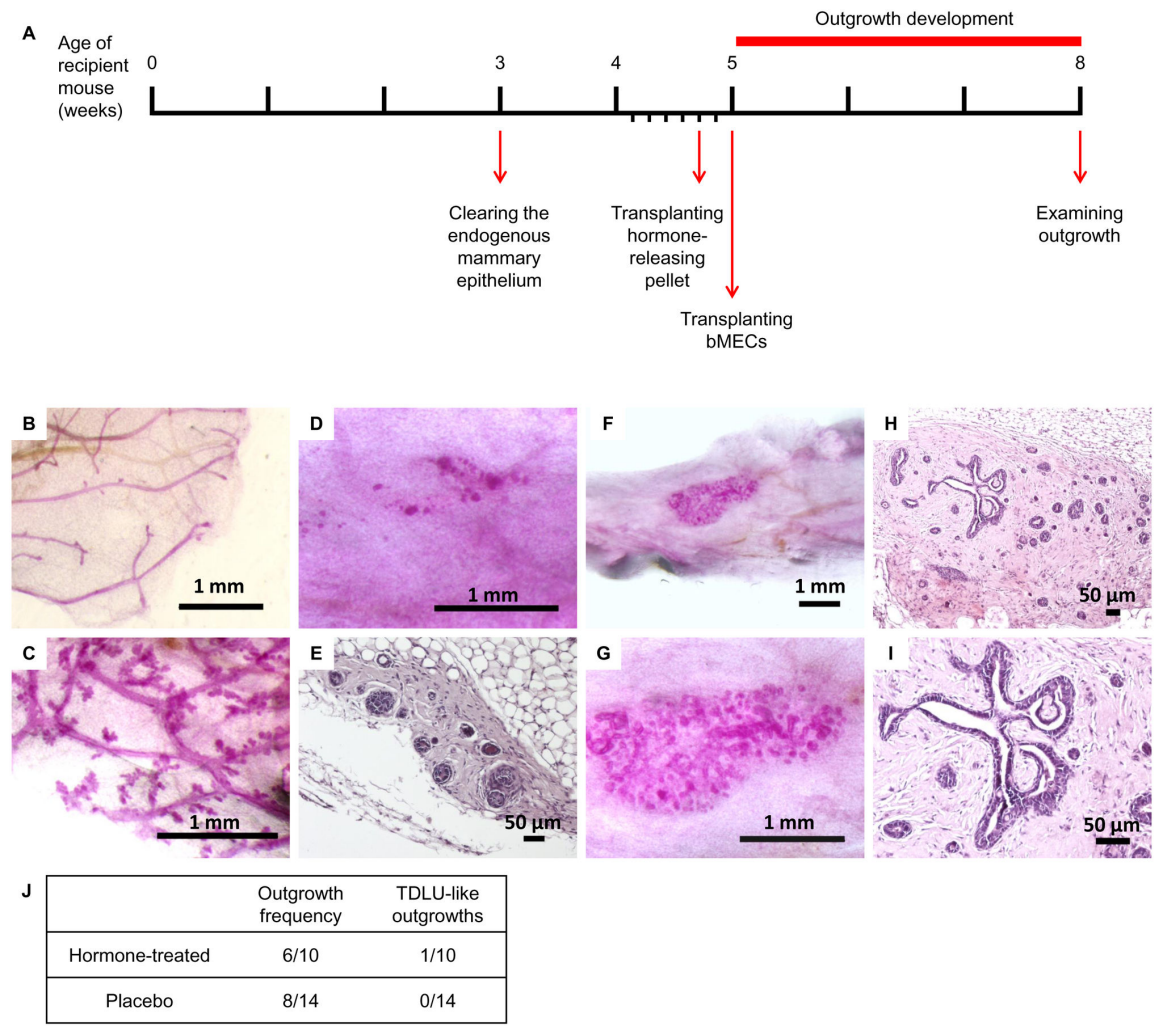
A TDLU-like structure develops from transplanted bovine cells in mice treated with estrogen and progesterone. **A**: Timeline of hormone treatment and bMEC transplantation. Hormone pellets were inserted 2 days prior to bMEC transplantation. **B**, **C**: Carmine-stained endogenous mammary epithelium from recipient mice carrying placebo (B) or hormone pellets (C). Enhanced branching of the endogenous ductal network is noted in (C). **D**, **E**: Carmine-stained wholemount (D) and H&E-stained paraffin section (E) depicting typical morphology of bovine outgrowth in the cleared mammary fat pad of hormone-treated mice. **F**–**I**: Carmine staining of mammary wholemounts (F, G) and H&E staining of paraffin sections (H, I), depicting a particular case of an outgrowth exhibiting significant growth and TDLU-like morphology under continuous systemic treatment of estrogen and progesterone. **J**: Frequency and morphology of outgrowths developed from transplanted bMECs in the cleared mammary fat pad of recipient mice that received, or did not receive, hormone treatment.

In an attempt to boost the development of bovine outgrowths in the foreign mouse environment, hormone-releasing pellets containing estrogen and progesterone were inserted subcutaneously into female NOD-SCID mice 2 days prior to bMEC transplantation, and outgrowths were examined 3 weeks later (Figure 3A). The systemic effect of the elevated steroid levels was confirmed by comparing Carmine-stained thoracic glands of host mice that had received placebo vs. those given the hormone-releasing pellets (Figure 3B and 3C). Enhanced branching and lobuloalveolar development were clearly visualized in the endogenous glands of the hormone-receiving group as compared to the short tertiary branching lacking lobuloalveolar development that characterized the placebo-receiving group. Ten mammary glands from mice transplanted with hormone pellets, and 14 mammary glands from mice transplanted with placebo were examined for the steroid hormones’ effect on outgrowth development. Figure 3D and 3E shows whole-mount and paraffin-section staining, respectively, of a representative outgrowth from the hormone-treated mice. The elevated levels of estrogen and progesterone in the circulation did not seem to affect outgrowth morphology or expansion: outgrowths remained restricted to the injection site, maintained limited growth and did not penetrate the mouse fat pad to reconstitute the full repertoire of bovine epithelial morphology. While this was the general case, one exceptional outgrowth was identified that exhibited extensive growth and formed duct-like structures resembling the bovine TDLU (Figure 3F–3I). This particular outgrowth developed in a fibrous microenvironment within the fat pad of the hormone-receiving host mouse, but as a single case; its dependence on elevated levels of estrogen and progesterone remains questionable. Finally, the effect of elevated estrogen and progesterone levels on bovine cell engraftment was monitored. Figure 3J demonstrates a comparable intake rate (60% and 57%) for cells transplanted to the cleared fat pads of hormone-treated and placebo mice, respectively. These results indicate that the steroid hormone treatment did not improve the bovine structures’ ability to occupy the mouse fat pad by either expansion or better intake.

### Co-transplanted mMECs establishes distinct mouse morphology and do not promote expansion capabilities of bovine outgrowths

Co-transplantation of normal mMECs with adult mouse testicular, neural or bone-marrow stem/progenitor cells [29–31], as well as with transformed mouse tumor cells or human embryonic carcinoma cells [32,33], induced reprogramming of the latter, promoting features of normal MECs and inhibiting tumorigenesis. Chimeric outgrowths developed with normal mouse mammary epithelial morphology, penetrating and expanding throughout the host adipose stroma.

**Figure 4:**
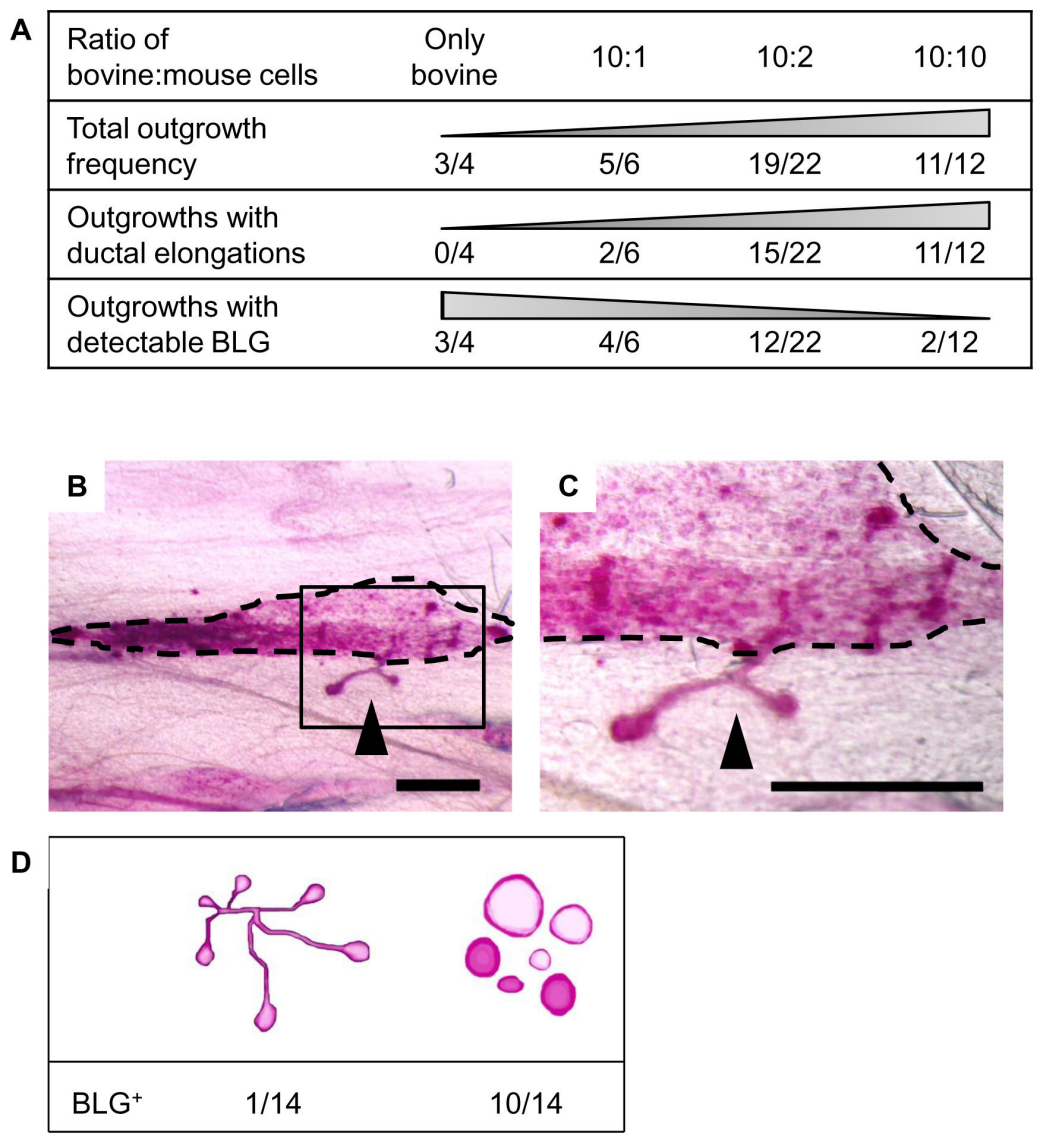
Co-transplantation of mouse and bovine epithelial cells rarely produces a chimeric outgrowth in the mouse. **A**: Frequency of outgrowths with mouse and bovine characteristics developed in the cleared mouse mammary fat pad. Outgrowths with ductal elongations and outgrowths with detectable DNA levels of the bovine-specific BLG sequences were monitored in fat pads transplanted with a range of bovine-to-mouse cell ratios. **B**, **C**: Low and high magnification, respectively, of Carmine-stained outgrowths developed from mouse and bovine MECs, co-transplanted at a ratio of 10:10. Region of spherical outgrowths is outlined in dashed line. Ductal protrusion is marked with arrowhead. Bar = 1 mm. **D**: Number of BLG-positive outgrowths developed in distinctly spherical and ductal outgrowth regions (n = 14 for each group).

To explore mMECs’ ability to promote bovine cell expansion within the host mouse’s stroma, a constant number of bMECs (4 x 10^5^) was transplanted alone, or with equal or lower numbers of mMECs (4 x 10^5^, 8 x 10^4^ or 4 x 10^4^). Outgrowth development was examined 6 weeks later. Bovine cell transplantation resulted in the development of typical confined and spherical structures in 75% of the transplanted glands (Figure 4A). In contrast, co-transplantation of bMECs and mMECs yielded outgrowths that contained two morphologically distinct structures (Figure 4B and 4C): confined spherical structures and elongated ducts with endbuds. The latter resembled the developing ductal network in mice [11]. Figure 4A demonstrates the inverse correlation between the proportion of bovine cells in the combined bovine/mouse cell mixture and overall outgrowth frequency. Outgrowth frequency ranged from 75% for transplantation of bovine cells alone to 91% for an equal mixture of bovine and mouse cells. A pronounced contribution of the mouse cells was shown for the development of outgrowths with ductal elongation (Figure 4A–4C). These morphological structures were not recorded in the absence of mMECs, and their frequency paralleled the proportion of mouse cells in the transplanted cell mixture. In transplants originating from equal numbers of bovine and mouse cells, outgrowths with ductal elongation developed in 91% of the glands.

The contribution of the bMECs to the outgrowth’s cell composition was verified by the presence of the bovine-specific BLG gene sequence in DNA isolated from the Carmine-stained outgrowth regions. Indeed, the development of outgrowths containing BLG sequences was positively correlated with the proportion of bovine cells in the transplanted bovine/mouse cell mixture (Figure 4A). Respectively, it decreased from 75% for bMEC-only transplantation to 16% in a mixture with an equal number of mMECs.

To compare bMEC incorporation into the two types of outgrowths, the spherical and ductal structures were separately dissected and individually analyzed for the presence of BLG sequences (n = 14 for each group; Figure 4D): 10 of the 14 spherical outgrowths were BLG-positive, whereas only 1 out of the 14 ductal outgrowths contained cells with detectable BLG sequences. Attempts to corroborate the genomic analysis with immunohistochemical definition of the individual cell types in the developing structures were performed by using anti-bovine nucleolin antibodies, or antibodies generated against a synthetic 10-aa peptide derived from a highly divergent region of the bovine NFκB protein sequence. Unfortunately, these antibodies did not react exclusively with the bovine cells. Nevertheless, the data indicate that bMECs are not a prominent constituent of the ductal outgrowths, and that co-transplantation with mMECs did not promote elongation or expansion of the structures composed primarily of bovine cells, which remained confined and spherical.

### Pre-transplantation of fibroblasts improves outgrowth frequency, but not expansion

**Figure 5:**
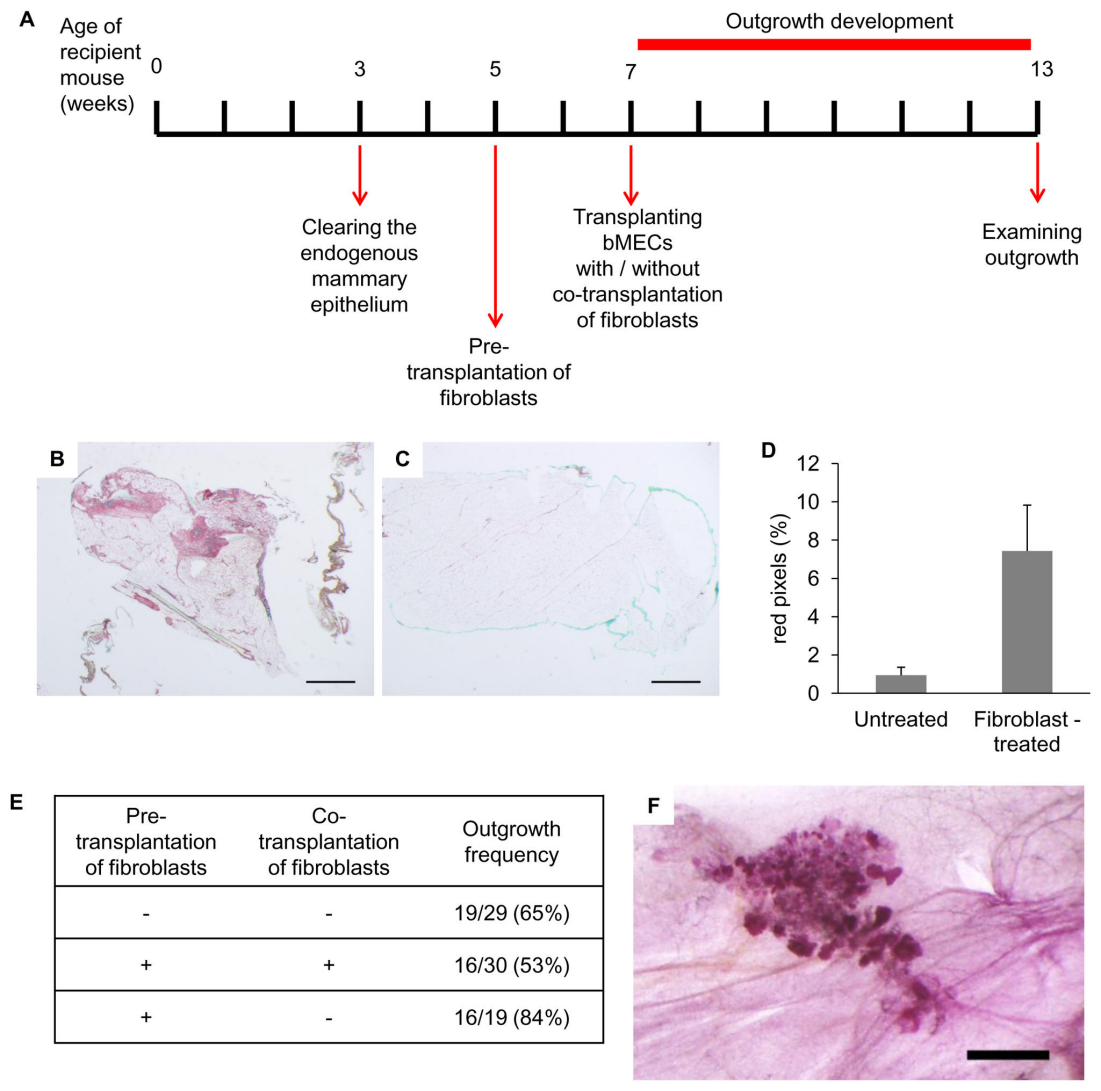
Differential effects for pre- and co-transplantation of fibroblasts on bMEC intake. **A**: Timeline of 10T1/2 fibroblast pre- and co-transplantation with bMECs. **B**, **C**: Representative Sirius red-stained tissue sections from cleared mammary fat pads, 2 weeks after fibroblast transplantation (B), or without intervention (C). **D**: The stained fibrotic area is significantly larger in fat pads transplanted with fibroblasts than in intact controls (*p* = 0.02, n = 5, error bars represent SEM). Percentage of red pixels was calculated using the Adobe Photoshop software. **E**: Effects of pre- and co-transplantation of fibroblasts on outgrowth frequency. **F**: Representative Carmine-stained wholemount of bovine outgrowth that developed within the cleared mammary fat pad 2 weeks after fibroblast transplantation. Bar = 1 mm.

The inability of the transplanted bovine and human MECs to initiate a developmental process that includes full occupancy of the cleared mouse mammary fat pad by differentiated duct-like and alveolus-like structures was intuitively associated with the more fibrotic composition of the bovine and human glands compared with the adipose mammary fat pad [34]. Indeed, pre- and co-transplantation of fibroblasts to the mouse fat pad have been shown to enable human ductal and lobular development [14,35]. The applicability of this approach for enhancing the frequency or expansion of bMEC outgrowths within the mouse fat pad was therefore tested by transplanting equal proportions of irradiated and non-irradiated 10T1/2 fibroblasts into the cleared mammary fat pads of NOD-SCID females, 2 weeks after clearing (Figure 5A). A change in stromal composition toward increased fibrosis was confirmed 2 weeks later by comparing fat pads from fibroblast-transplanted and non-transplanted mice (n = 5 for each group). Figure 5B and 5C demonstrates a larger Sirius red-stained area (indicating collagen) in the transplanted fat pads than in the intact mammary glands. Quantification of the stained areas in the two groups showed a sevenfold increase in the percentage of fibrous area following fibroblast transplantation (Figure 5D). At that time point, 2 weeks after fibroblast transplantation, bMECs were transplanted along with equal numbers of irradiated and non-irradiated 10T1/2 fibroblasts (Figure 5A). It should be noted that subjecting fibroblasts to irradiation results in an active state, thus facilitating engraftment of the stromal cells [35].

To monitor any direct effects of the fibroblasts on bMEC fate and function [29,30], a control group that did not receive the second fibroblast transplant was established. A second control group was transplanted only with bMECs and not with fibroblasts. Outgrowths were examined 6 weeks after bMEC transplantation.

Pre- and co-transplantation of 10T1/2 fibroblasts did not significantly (*p* < 0.05, two-proportion Z test) affect outgrowth frequency compared with the untreated group (Figure 5E). However, when fibroblasts were transplanted only before bMEC transplantation, outgrowth frequency was significantly (*p* = 0.01) improved (by 31%) compared with the group exposed to both pre- and co-transplantation. An improvement of 19% was also detected relative to the group that was not transplanted with fibroblasts (*p* = 0.06). Finally, transplantation of 10T1/2 fibroblasts did not affect outgrowth morphology, which remained spherical and confined (Figure 5F).

### Adipose tissue-conditioned medium inhibits mammosphere development from dispersed bMECs in culture

Data from the earlier experiments (Figure 1) suggested that the mouse mammary adipose stroma does not support the development of bovine mammary epithelium in the host mouse. We sought to determine whether this involves active growth inhibition, and to distinguish between the putative negative effect(s) mediated by cell–cell interactions and a paracrine effect.

3D mammosphere culture is an established *in vitro* model that recapitulates basic developmental and functional properties of the mammary gland [36,37]. It enables growth of mammary epithelia without stromal interaction. Here, mammosphere development within Matrigel from dispersed bMECs was monitored for 3 days in the presence of adipose-conditioned medium, previously incubated with explants of mouse mammary adipose tissue for 6 days. Respective controls were: (i) fresh (unconditioned) medium; (ii) bMEC-exhausted medium, previously incubated with epithelial cells for 6 days. The mammary adipose explants were derived from the inguinal mammary fat pad of 3-month-old wild-type female mice which was cleared of its epithelial component. Explant number and culture duration were designed to minimize the usage-degree effect of the supplemented media, which was monitored according to ammonia accumulation. In culture, ammonia accumulates during amino-acid metabolism and also due to decomposition of the glutamine added to the medium [38].

**Figure 6:**
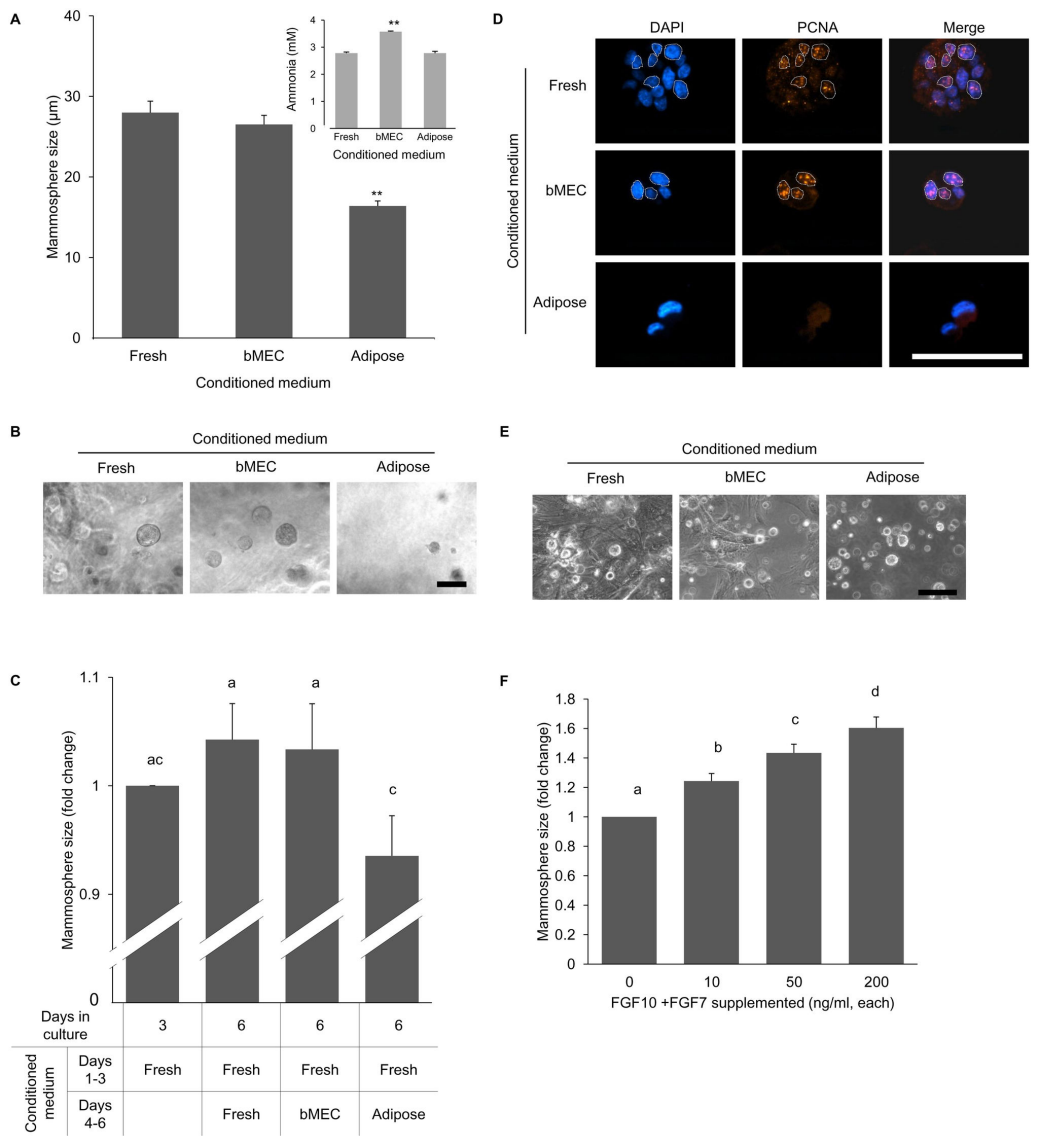
Growth of bovine epithelial mammospheres is suppressed by adipose-conditioned medium and rescued by FGF supplementation. **A**: Mammospheres formed by bMECs cultured for 3 days within Matrigel are smaller when supplemented with adipose-conditioned medium as compared to bMEC-conditioned or fresh medium. Columns represent average size ± SEM of 28 mammospheres measured in each group. Results of one representative experiment out of three biological replicates are presented. Inset: ammonia levels accumulated in adipose-conditioned, bMEC-conditioned and fresh media. Columns represent average size ± SEM from four analyses of each medium. **Statistically significant difference (*p* ≤ 0.01) compared to other columns. **B**: Light microscopy demonstrating the effect of the culture medium on the size of the developing mammospheres. Bar = 50 µm. **C**: The effect of the culture medium (fold change) on the size of 3-day-developed mammospheres. Columns represent average size ± SEM of 60 mammospheres measured in each group. Different letters above the columns represent statistically significant differences (*p* ≤ 0.05). **D**: Immunofluorescent staining of PCNA in representative mammospheres formed by bMECs in fresh and conditioned media. PCNA-positive cells are encircled by a white dotted line. Bar = 50 µm. **E**: Light microscopy demonstrating no effect of adipose-conditioned medium on the size of mammospheres formed by mMECs. Absence of fibroblasts in cultures supplemented with adipose-conditioned medium is noted. Bar = 100 µm. **F**: FGF7 and FGF10 supplementation to the adipose-conditioned medium induces mammosphere size increase in a dose-dependent manner. Columns represent average size ± SEM of 60 mammospheres measured in each group. Different letters above the columns represent statistically significant differences (*p* ≤ 0.05).

Adipose-conditioned medium negatively affected bovine mammosphere development *in vitro*, as manifested by their smaller size—approximately 40% of those developed in fresh, or bMEC-conditioned medium (Figure 6A and 6B). This was independent of the degree of medium usage, which was even higher in the bMEC-conditioned medium (Figure 6A, inset). It should be noted that the effect of adipose-conditioned medium derived from explants of younger females (3 weeks old) was markedly lower, causing a decrease of only 13% in mammosphere size (data not shown). This difference may be attributed to the changes in global gene-expression profile in mouse mammary stroma during puberty [39] and to mammary adipose differentiation and maturation [40,41], which are evident to the naked eye as a higher amount of fat droplets generated by explants from mature compared to 3-week-old mice.

The inhibitory effect of adipose tissue was further analyzed on developed mammospheres, supplemented for the first 3 days of culture with fresh medium (Figure 6C). These mammospheres were then treated with adipose-conditioned medium for another 3 days. For controls, mammosphere growth medium was replaced with either bMEC-conditioned or fresh medium. In the control cultures, mammospheres continued to grow during days 4–6 of culture. In contrast, supplementation of adipose-conditioned medium resulted in growth arrest, as depicted by the comparable size of the mammosphere at the beginning and end of the second incubation period. No significant differences (*p* < 0.05) were detected between the inhibitory effects of conditioned medium from adipose explants prepared from cleared vs. uncleared fat pad (data not shown).

PCNA immunostaining was performed to mark proliferating cells within the mammospheres (Figure 6D); 11.4 ± 1.8% and 18.6 ± 3.8% of the cells in mammospheres grown in fresh and bMEC-conditioned media were PCNA-positive, respectively. In contrast, when supplemented with adipose-conditioned medium, no PCNA-positive cells could be detected within the mammospheres (n = 5 fields and at least 28 cells tested in each group).

The results of these experiments confirmed that mouse mammary adipose stroma exerts a negative effect on the development of mammospheres from bMECs *in vitro*, and that this effect does not require cell–cell contact.

Further analysis was aimed at determining the effect of mouse adipose-conditioned medium on mammosphere development from epithelial cells of the same species. Mouse adipose-conditioned medium did not affect the growth of mammospheres from dispersed mouse epithelial cells *in vitro*, when compared with fresh medium or bMEC-conditioned medium (Figure 6E). However, it eliminated the growth of accompanying fibroblast-like cells. Given the tendency of bovine outgrowths to thrive in fibrotic regions, the negative effect of mouse adipose-conditioned media on fibroblast growth might participate in the mechanism by which the mouse stroma hinders the development of bovine mammary epithelial outgrowths.

Fibroblast growth factors (FGFs) and their receptors play a pivotal role in the development of mammalian branched systems through interactions between the epithelia and the stroma [5,42]. In the mouse mammary gland, FGFs regulate cell proliferation and survival in the terminal endbud [43]. FGF10 and FGF7 are produced in the mammary stroma [44]. Together with their receptor FGFR2, which is located in the epithelium [42], they are of particular importance for mammary embryonic as well as postnatal development and morphogenesis [45–48].

Possible intervention of FGF signaling along with the negative effect of the mouse stroma was studied by adding FGF7 and FGF10 at increasing concentrations to the inhibitory adipocyte-conditioned medium. Mammosphere size was measured after 3 days of culture. Supplementation of FGF7 and FGF10 induced a dose-dependent increase in mammosphere size (Figure 6F), thus overcoming the suppressive effect of the adipose-conditioned medium.

### Mouse mammary adipose-conditioned medium inhibits the development of human breast mammospheres

**Figure 7:**
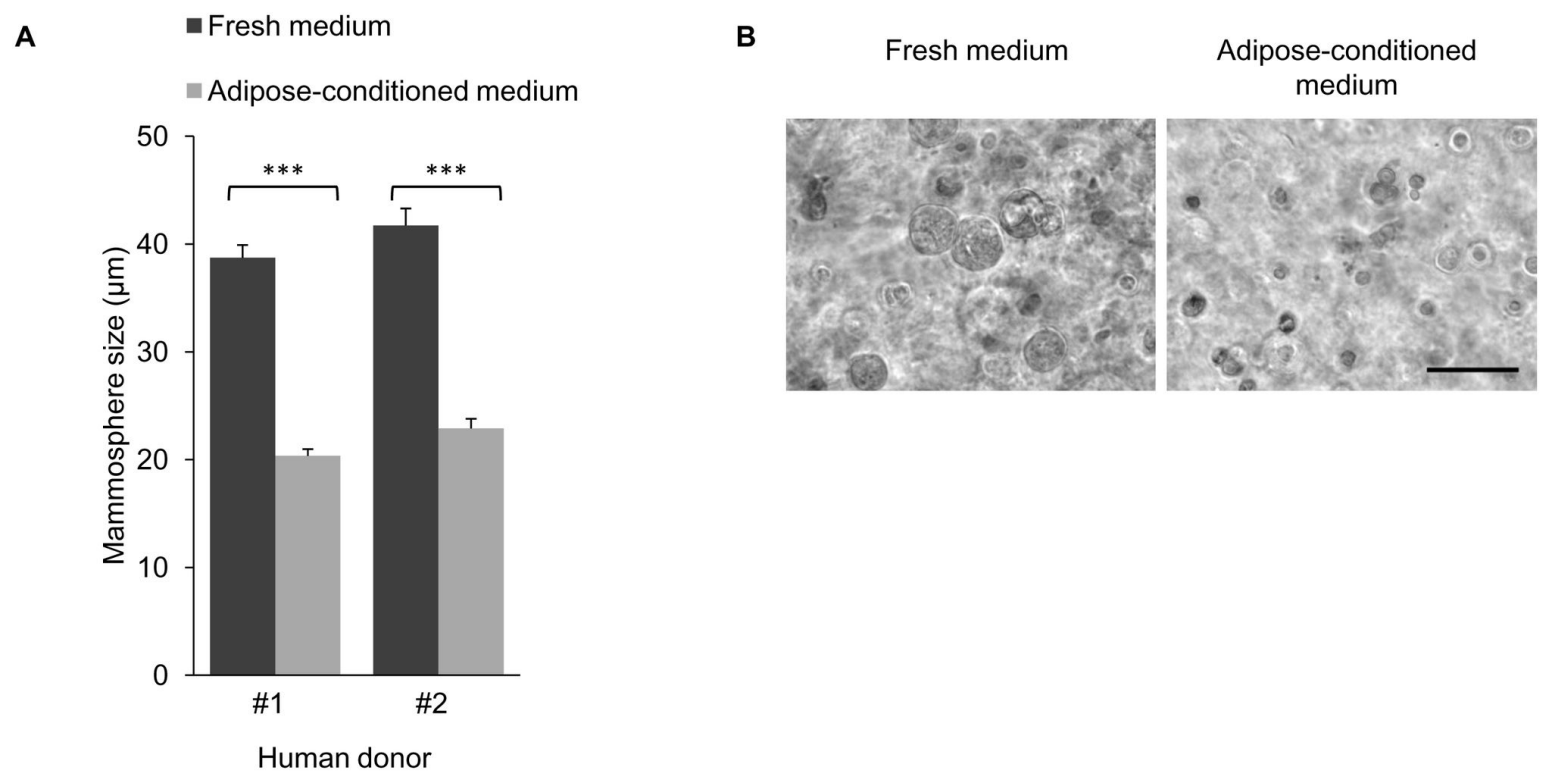
Adipose-conditioned medium suppresses the development of human breast mammospheres in culture. **A**: Average size of mammospheres formed by hMECs from the breast tissue of two donors after 3 days of culture in fresh or adipocyte-conditioned medium. Columns represent average size ± SEM of 60 mammospheres measured in each group. ***Statistically significant difference (*p* ≤ 0.001). **B**: Light microscopy of representative mammospheres formed by hMECs in 3D Matrigel culture with fresh or adipose-conditioned medium. Bar = 100 µm.

The development of human- and bovine-representative mammary epithelial morphology is subjected to a comparable inhibitory effect when transplanted into the mouse cleared mammary fat pad [13]. To test whether the inhibition exerted by the mouse adipocyte-conditioned medium also affects human mammosphere development, it was supplemented for 3 days into 3D cultures of dispersed epithelial cells, derived from the breasts of two donors. Addition of the mouse adipocyte-conditioned medium to the human breast cell culture inhibited the growth of mammospheres from both donors by ~50%, as compared to mammospheres developed in fresh medium (Figure 7A and 7B).

Taken together, these experiments demonstrate a comparable inhibitory effect of the mouse adipose-conditioned medium on bovine and human mammosphere development, but not on the development of mouse mammospheres. The data may therefore attest to the *in vivo* limitation in the development of human and bovine representative mammary morphology within the mouse fat pad, which contrasts with the expansion of mouse outgrowth in the same environment.

## Discussion

Transplantation of bMECs into the mouse mammary stroma results in only partial reproduction of bovine mammary gland morphology. Three possible reasons for this were addressed in this study: i) residual immune response; ii) insufficient hormonal stimulus, and iii) inappropriate stromal environment.

To minimize the host immune response which might affect bovine epithelial development in athymic nude mice [12], bMECs were transplanted into the cleared mammary fat pad of NOD-SCID mice. NOD-SCID mice lack T and B cells, and have impaired natural killer (NK) cell immunity. In contrast, athymic nude mice lack T cells, but maintain functional B cells as well as NK cell immunity [49]. The bovine epithelial outgrowths did not seem to expand any further in the NOD-SCID hosts than in the athymic nude mice, suggesting that a putative residual immune response is not a major reason for their limited development. Nevertheless, the growth of the spheres in the more accommodating NOD-SCID immunocompromised mice was not restricted by the 6-week growth barrier reported for transplanted athymic nude mice [34]. Indeed, by 6 weeks, the hollow structures that developed in the NOD-SCID mouse fat pad maintained actively proliferating epithelial cells that supported their continuous growth. Histological and immunohistochemical characterization indicated that the spheres resemble the bovine alveoli in terms of size, orientation of keratin expression, ER/PR localization and expression pattern, and cell functionality, as depicted by their capacity to synthesize milk proteins. Importantly, the absence of an apparent "draining" ductal network suggests at least a delay in the hierarchical process that involves specific differentiation of stem cells via putative ductal-specific progenitors [50].

The pre-pubertal effect of estrogen and progesterone on elongation and branching of the mammary ductal network was enhanced using slow-release pellets, as opposed to the daily subcutaneous injections performed by Sheffield and Welsch [12]. The latter procedure increased^3^ [H] thymidine intake in the outgrowth by 167%, with no notable effect on its morphology. Here, the continuous administration of steroid hormones induced further expansion and branching of the endogenous rudimentary mouse ductal network, but had an irreproducible effect on the bovine epithelial morphology, as reflected by the development of a single TDLU-like structure, out of 14 transplants. Morphological and functional changes have been detected in the bovine gland after administration of comparable or lower levels of progesterone and estrogen per equivalent body weight, respectively [51,52]. It is therefore unlikely that insufficient levels of steroid hormone *per se* caused the inconclusive effect of estrogen and progesterone on the morphology of the bovine outgrowths.

Regardless of this developmental drawback, establishing the feasibility of such advanced development is an important starting point for further development of better conditions to support the growth of foreign epithelium. Indeed, adequate cross-talk between the stroma and the hormone-responsive epithelial cells is required for steroid hormone activity in the mouse mammary gland [42,53–56]. Paracrine signaling of estrogen and progesterone has also been proposed in the bovine mammary gland [23] and the human breast [57,58]. However, due to a lack of models for knockout and tissue-recombination experiments, these could not be fully confirmed.

The notion that an unsuitable stromal environment hinders the expansion of human mammary outgrowths in mice, coupled with the potential contribution of a successful xenotransplantation model to breast cancer research, has prompted attempts to enhance the limited growth and development of human (h) MECs in mice. Progress has been achieved by adapting the stromal composition of the host fat pad to resemble the more fibrous human one [14,35]. The most frequently observed outgrowths remained acinar spherical structures with hollow lumina. However, ductal and lobular structures also developed. Consequently, the current report represents the first attempt to allow the development of transplanted bovine epithelia by increasing the fibrous content of the mouse mammary gland via pre- and co-transplantation of fibroblasts. Indeed, a more fibrous stroma was generated and the intake rate of bovine cells increased after fibroblast pre-transplantation. However, this procedure did not cause expansion of the bovine outgrowth in the mouse cleared mammary fat pad or the generation of TDLU structures.

Methodological reasons, such as the transplantation of human organoids vs. dispersed bMECs, or the type of fibroblasts used, might account for the lack of comparable developmental progress of the transplanted bovine epithelial cells. However, recent studies have involved transplantation of dispersed human cell populations [59,60] and comparable effects of fibroblasts from different sources have been demonstrated [61]. It could be that looser intrinsic control of growth occurs in the transplanted breast cells compared to the bovine ones. This could be reflected by the absence of apparent malignant development from transplanted bovine cells (of 10 different donors, our observation) compared to hyperplasias and tumors developed in the same immunodeficient mice after direct human organoid transplantion [14].

The third approach to facilitating the development of representative bovine mammary structures in the cleared mouse fat pad involved the generation of chimeric bovine–mouse outgrowths. Developmental signals from the microenvironment, generated by the engrafted mouse mammary cells, induced reprogramming of non-mammary and tumorigenic cells to adopt a normal mammary fate [19,62]. Here, the chimeric bovine–mouse model failed to support representative bovine mammary structural morphology in the cleared mouse fat pad. The outgrowths that branched and penetrated the stroma exhibited a clear mouse epithelial morphology, manifested by terminal endbuds as opposed to the bovine TDLU. Only a single outgrowth (out of 14) contained detectable bovine-specific BLG DNA sequences. It cannot be ruled out that few bMECs might have elongated along with mMECs in other outgrowths and escaped BLG DNA detection. Technical difficulties prevented their identification by immunohistochemistry with antibodies generated against selected bovine proteins. The mechanism via which the newly generated niche, created by the chimeric transplantation, affects foreign cell fate is unknown. Competition for a similar cell-signaling pathway might be involved [19,62]. Nevertheless, the events leading to alteration of the cell’s fate are likely to occur early on, shortly after the transplantation. We postulate that at this early stage, bovine cells are designated to either participate in the formation of mouse-shaped epithelium or to generate spherical outgrowths. We saw no evidence of an intermediate program, in which the spherical bovine outgrowths adopt features of mouse morphology such as the ability to form ducts and penetrate the stroma. An insufficient number of cells with undetermined fate in the heifer’s gland might be the cause of the inefficient reprogramming of the bovine cells compared to neural stem cells, bone-marrow cells or those composing the male seminiferous tubules.

The developmental limitation of the bovine outgrowths in the mouse fat pad could be graded according to the site of cell integration. Multilayered proliferating structures developed within fibrotic areas and unilayered round structures were located at the border between the fibrotic area and the adipocytes. The most severe phenotype of scattered epithelial cells with no apparent interactions was found in the adipocytes. *In vitro* analysis of a cultured mammosphere model revealed that not only is the mouse adipose stroma unable to support the growth of mammary epithelial structures from bovine and human species [34], it actively inhibits it. We hypothesize that the inhibition is mediated by a factor(s) secreted from the mouse fat pad that interferes with the proliferation of the cells composing the mammospheres. However, a negative effect due to consumption of individual essential factors, from either the medium or the immediate microenvironment, that affect foreign epithelial outgrowth cannot be excluded.

An inhibitory role for adipocytes has been demonstrated on hematopoietic progenitor expansion within the adipocyte-rich bone marrow, and mice genetically deficient in adipogenesis showed accelerated hematopoietic recovery after bone-marrow ablation [63]. In the mammary gland, limitation of the endogenous expansion, branching and cell proliferation of the epithelial component has been associated with fat deposition in bovines [64,65], and obesity in mice [66]. Cross-species inhibition of the proliferation of a cultured human MCF7 breast cancer cell line by conditioned medium of 3T3-L1 mouse pre-adipocytes was also demonstrated [67]. In the latter experiments, the inhibitory mechanism was associated with the effect of a secreted protein that does not correspond to the characteristics of IL-1, IFN-γ, IFN-α, TNF-α or TGF-β.

To this end, an attractive candidate for mediation of the growth inhibition exerted by adipocytes on the development of foreign epithelia is the Stat3 axis, which funnels the repressive effect of adipocyte-secreted leptin and IL-6 on the expansion of mammary epithelium [68–71]. Leptin expression increases threefold during sexual maturation [39] and correlates with the higher inhibitory effect of conditioned medium from mammary adipose explants of mature vs. 3-week-old females. Since intact leptin signaling through STAT3 is also essential for lactation [72], it seems that the responsiveness of the mammary epithelial cells to leptin, IL-6 or other potential paracrine inhibitors is determined by the levels of these factors in the vicinity of the cells, as well as by receptor number and affinity. Due to the *in-vivo* proximity of mMECs to stromal adipocytes, these cells may be inherently less sensitive to this inhibition than bovine or human MECs, which are separated and located further away from the adipose stroma. Further studies are warranted to characterize the activities of those adipocytes which are in close contact with the transplanted cells.

The accommodating fibrotic stroma may not only protect the developing foreign epithelium from the adipose inhibitory effect, it may also generate the opposing activity of antagonizing signals. FGF10 and FGF7 are secreted from the mammary stroma and are required for epithelial morphogenesis [5,73]. In culture, these FGFs rescue mammosphere growth from adipocyte inhibition. By augmenting AKT/PKB-induced proliferation, FGFs may antagonize Stat3 involvement in mammary involution [74–76]. Alternatively, they could induce cell proliferation via the β-catenin pathway.

Taken together, the data accumulated in this study lead to our hypothesis that the mouse fatty stroma serves as a local line of defense which, via the adipocyte paracrine effect, actively inhibits the development and particularly the expansion of foreign epithelia. Conditions that simulate the endogenous microenvironment of the bovine epithelium and support its development can be exerted by enhancing positive signals such as those generated by the FGFs.

## Materials and Methods

### Immunodeficient mice

NOD-SCID mice were purchased from Harlan Laboratories (Jerusalem, Israel). They were kept in sterilized cages and supplemented with sterile water and irradiated food *ad libitum*. For all surgical procedures, mice were anesthetized with isoflurane (Abbott Laboratories, Maidenhead, England) mixed with O_2_ using a veterinary anesthesia machine. All animals used in this study were treated humanely. Study protocols were in compliance with the regulations of the Israeli Ministry of Health and the Animal Experimentation Ethics Committee of the Agricultural Research Organization, The Volcani Center (approval no. IL- 202/09).

### Dissociation of bovine mammary tissue into single-cell suspension

Bovine mammary tissue was harvested and dissociated into a single-cell suspension as previously described [24]. Briefly, the parenchymatic region was excised from the udder of 7- to 10-month-old Holstein heifers that had been commercially slaughtered. The tissue was mechanically minced and enzymatically digested by collagenase and hyaluronidase into organoids which were aliquoted and stored at -80°C. During this procedure, the dissociated adipose tissue floats over the supernatant and is subsequently discarded. Upon use, organoids were further dissociated by trypsin (Biological Industries, Beit Haemek, Israel) and dispase (BD Biosciences, Bedford, MA) to yield a single-cell suspension of bMECs.

### bMEC transplantations

The endogenous mammary epithelium was surgically removed from the 4^th^ mammary glands of 21-day-old NOD-SCID females mice of <10 g body weight and before the growing ducts reached the lymph node (i.e. "clearing" [11,20,77]); 4 weeks later bMECs were transplanted into the cleared fat pad. When hormonal administration was involved, bMECs were transplanted 2 weeks after clearing, when the mice were 5 weeks old. The cells were suspended in 20 µl Matrigel (BD Biosciences) diluted 1:1 (v/v) in HF solution containing Hank’s balanced salt solution (HBSS, Biological Industries) supplemented with 0.1% Hepes (Biological Industries) and 2% fetal bovine serum (FBS, Biological Industries). Cell suspension in Matrigel was injected into the cleared mammary fat pad using a 50-µl Hamilton syringe (Hamilton Company, Reno, NV) equipped with a 21-gauge needle. Unless otherwise indicated, outgrowths were allowed to develop for 6 weeks before the transplanted fat pad was removed and visualized. This period was shortened to 3 weeks in experiments involving steroid hormone administration due to some mortality observed beyond this time point.

### Hormone administration

Two days prior to the bMEC transplantation, slow-release hormone pellets containing 1.7 mg 17β-estradiol and 16.7 mg progesterone (Innovative Research of America, Saratosa, FL) were inserted subcutaneously in the mouse’s nape region, according to the manufacturer’s protocol.

### Co-transplantation of mouse and bovine MECs

Mouse cells were dissociated from the 4^th^ inguinal mammary glands of 6- to 8-week-old FVB/N virgin females. The dissociation procedure for the mouse mammary gland followed that for the bovine tissue, except that the enzymatic digestion was shortened to 1 h. Single-cell suspension of bovine cells was obtained as described above. Mouse and bovine cells were mixed and suspended at the indicated ratios in 20 µl Matrigel, diluted 1:1 in HF solution and transplanted into the cleared mammary fat pad of NOD-SCID mice.

### Pre and co-transplantation of fibroblasts with bMECs

Fibroblasts of line 10T1/2 (kindly provided by Eyal Bengal, The Rappaport Institute, Technion Israel Institute of Technology, Haifa, Israel) [78] were cultured in DMEM supplemented with 10% FBS, glutamine (365 µg/ml), gentamicin (50 µg/ml), streptomycin (100 µg/ml), and penicillin (100 U/ml, all from Biological Industries). This medium is referred to as "fibroblast medium". Upon semi-confluence, cells were trypsinized, harvested, centrifuged and resuspended in fibroblast medium. Half of the cells were irradiated at 50 Gy in conical tubes containing up to 10^7^ cells/ml in fibroblast medium. A 1:1 mixture of irradiated and untreated fibroblasts (25 x 10^5^ cells each) in 20 µl of 1:1 HF solution: Matrigel were transplanted into the cleared mammary fat pad of NOD-SCID females 2 weeks before bMEC transplantation. For co-transplantation of fibroblasts and bMECs, a 1:1 mixture of irradiated and intact fibroblasts (25 x 10^5^ cells each) was combined with bMECs (1 x 10^6^). Cells were resuspended in 20 µl of 1:1 HF: Matrigel and transplanted into the cleared mammary gland of NOD-SCID mice as described above.

### Outgrowth analysis

For whole-mount examination, the transplanted mammary fat pads were excised from sacrificed mice and fixed on glass slides with 4% paraformaldehyde containing 1% sucrose for 2 h at room temperature. Whole mounts were washed with PBS and stained with Carmine-alum (Sigma) overnight at room temperature as previously described [79]. Whole mounts were dehydrated with ethanol and cleared in xylene or K-clear reagent (Kaltek, Padova, Italy) overnight at room temperature, and then visualized and photographed using a binocular (Olympus SZX16, Tokyo, Japan) equipped with CellSens standard 1.4 software (Olympus). The tissues were then embedded in paraffin blocks for further analyses.

### Histological analyses and immunohistochemistry

Histochemical analyses of outgrowths, including immunostaining, were performed on 5-µm paraffin sections dissected from the Carmine-alum-stained whole mounts. The Carmine staining procedure was necessary to distinguish the small areas of outgrowths within the fat pad, and for their extraction. Paraffin sections were stained with H&E (Sigma) to visualize the morphology of the epithelial structures, or with Sirius red (Sigma) to visualize the fibrotic areas, marked by the collagen staining. For immunostaining of the outgrowths and the bovine mammary tissue, sections were incubated with selected primary antibodies (Table 1), washed and reacted with HRP-labeled anti-mouse and anti-rabbit secondary antibody (N-Histofine, Nichirei Biosciences, Tokyo, Japan). Signals were generated with DAB substrate (Vector Laboratories, Burlingame, CA).

**Table 1:** List of primary antibodies applied in the immunoblot analyses.

**Antigen**	**Antibody**	**Dilution**	**Manufacturer**
PCNA	Mouse monoclonal, clone PC10	1:200	BioLegend, San Diego, CA
Cytokeratin 14	Mouse monoclonal, clone LL002	1:200	AbD Serotec, Oxford, UK
Estrogen receptor α	Rabbit polyclonal	1:50	Santa Cruz Biotechnology
Progesterone receptor	Mouse monoclonal, clone αPR6	1:50	Abcam
β-Lactoglobulin	Rabbit polyclonal	1:50	Nordic Immunological Laboratories, Tilburg, The Netherlands
β-Casein	Rabbit polyclonal	1:50	[81]

3D Matrigel cultures were histochemically analyzed after fixation in Bouin’s solution, dehydration in a graded ethanol series (50% to 100%), clearing in xylene and paraffin embedding. Paraffin sections (5-µm thick) were stained with H&E, or reacted with PCNA primary antibody (Table 1) followed by a secondary antibody (Cy3-conjugated donkey anti-mouse IgG, Jackson ImmunoResearch, West Grove, PA). Nuclei were visualized by DAPI staining (10 µg/ml, Santa Cruz Biotechnology, Santa Cruz, CA). Sections were mounted with Pro-Long mounting reagent (Molecular Probes, Eugene, OR). Stained sections were visualized and photographed by an inverted fluorescence microscope (Eclipse Ti, Nikon Instruments, Melville, NY) equipped with NIS-Elements AR 3.2 imaging software (Nikon Instruments).

### Isolation of genomic DNA from outgrowths and detection of BLG sequences by RT-PCR

Genomic DNA was isolated from paraffin sections of outgrowths using the QIAmp DNA FFPE tissue kit (Qiagen, Hilden, Germany) according to the manufacturer’s protocol. Quantitative real-time PCR analyses were performed in a StepOnePlus instrument (Applied Biosystems, Foster City, CA) in a 20-µl reaction volume containing 5 µl DNA, 10 µl SYBR Green fast PCR Master Mix (Applied Biosystems) and 10 mM primers. BLG primers (forward 3'-TGCTGGACACCGACTACAA-5', reverse 3'-TCAGCACTGTTCTCCATGC-5') and 16S primers (forward 3'-TCGATGTTGGATCAGGACA-5', reverse 3'-AATCGTTGAACAAACGAACC-5') were used. The thermal-cycling conditions consisted of 20 s at 95°C followed by 40 cycles of 3 s at 95°C and 30 s at 60°C. PCR of DNA isolated from bovine mammary tissue yielded a single product with a peak melting point at 73°C. PCR of mouse mammary tissue yielded several irreproducible by-products, none of which had a peak melting point at 73°C, indicating lack of BLG gene in these samples. All samples were analyzed in triplicate.

### 3D cell cultures

Cells from three mammalian species were cultured in 3D culture conditions: i) bMECs, ii) mMECs and iii) hMECs. bMECs were dissociated from organoids as described above. mMECs were dissociated from a pool of eight freshly harvested no. 4 mammary glands, excised from 3-month-old wild-type FVB/N virgin mice, as described above. hMECs were dissociated from frozen organoids prepared from fresh human breast tissue donated by two patients who had undergone elective surgery for breast implantation replacement at Kaplan Hospital, Rehovot, Israel. Donor 1 was 25 years old with no prior pregnancies; donor 2 was 37 years old with three prior pregnancies. All human breast tissue procurement for these experiments was in compliance with laws and guidelines approved by the Helsinki Institutional Review Board committee of Kaplan Hospital. Participants provided a written consent to participate in this study. Enzymatic digestion of the human breast tissue to organoids, as well as dissociation of hMECs from organoids, were performed as described for bMECs.

Dispersed bMECs and hMECs were cultured for 6–7 days, avoiding confluence, in mammary medium [24] composed of DMEM-F12 medium supplemented with 5% FBS, hydrocortisone (0.5 µg/ml, Sigma), insulin (5 µg/ml, Sigma), gentamicin (50 µg/ml, Biological Industries), streptomycin and penicillin (100 µg/ml and 100 U/ml, respectively, Biological Industries), hEGF (10 ng/ml, Merck, Darmstadt, Germany), hFGF2 (10 ng/ml, Merck), heparin (4 µg/ml, Merck), cholera toxin (10 ng/ml, Sigma) and B27 (4 ml stock/ml medium, Invitrogen, Carlsbad, CA). Cells were then harvested by trypsinization and resuspended in 1:1 DMEM: Matrigel at a concentration of 1000 cells/µl; 50-µl droplets, each containing 50 x 10^3^ cells, were carefully placed at the bottom of wells in 24-well plates and supplemented with 0.5 ml conditioned or fresh mammary medium for 3 days, unless otherwise indicated. Adipose-conditioned medium was collected from 6-day explant cultures [80] of cleared mouse mammary adipose tissue in mammary medium (six explants of 1–2 mm^3^/ml). When indicated, it was supplemented with hFGF7 and hFGF10 (Peprotech, Rehovot, Israel). bMEC-conditioned medium was obtained from 6-day cultures of freshly dissociated bMECs in mammary medium. Ammonia levels in the conditioned media were monitored during culture using EnzyChrom Ammonia assay kit (ENH3-100, BioAssay Systems, Hayward, CA), according to the manufacturer’s protocol. 3D cultures were visualized after 3 days by light microscopy (Eclipse Ti, Nikon Instruments) and mammosphere size was measured using NIS-Elements AR 3.2 software (Nikon Instruments). At least 60 mammospheres were measured in each group. 3D Matrigel cultures were then fixed in Bouin’s solution and processed for histological and histochemical analyses as described above.
